# TFAP2A‐Induced Upregulation of LncRNA NUTM2A‐AS1 Promotes LUAD Progression Through a miR‐409‐5p/SLC35F2 Regulatory Axis

**DOI:** 10.1111/jcmm.71284

**Published:** 2026-07-10

**Authors:** Weiqin Wang, Yongfeng Liu, Tiantian Chen, Jing Zhang, Feng Hu

**Affiliations:** ^1^ Department of Respiratory and Critical Care Medicine Tongren Hospital, Shanghai Jiao Tong University School of Medicine Shanghai China

**Keywords:** LncRNA, lung adenocarcinoma, miRNA, SLC35F2, TFAP2A

## Abstract

Long non‐coding RNAs (LncRNAs) play pivotal roles in lung adenocarcinoma (LUAD) progression. However, the upstream transcriptional regulation and downstream mechanisms of LncRNA NUTM2A‐AS1 in LUAD remain largely unexplored. The expression levels of NUTM2A‐AS1, TFAP2A, miR‐409‐5p and SLC35F2 in LUAD tissues and cell lines were analysed using public databases and qRT‐PCR. The biological functions of this axis in LUAD cells were evaluated through CCK‐8, Transwell and apoptosis assays. The molecular interactions among TFAP2A, NUTM2A‐AS1, miR‐409‐5p and SLC35F2 were validated via chromatin immunoprecipitation (ChIP), dual‐luciferase reporter and RNA immunoprecipitation (RIP) assays. Both NUTM2A‐AS1 and TFAP2A were significantly upregulated in LUAD and correlated with poor prognosis. Mechanistically, the transcription factor TFAP2A directly bound to the NUTM2A‐AS1 promoter to activate its transcription. Functionally, NUTM2A‐AS1 promoted LUAD cell proliferation, migration, invasion and epithelial‐mesenchymal transition (EMT), while suppressing apoptosis. Furthermore, NUTM2A‐AS1 may function as a competing endogenous RNA‐like regulator by interacting with miR‐409‐5p, thereby upregulating the expression of the solute carrier family member SLC35F2. Rescue assays demonstrated that SLC35F2 knockdown attenuated the oncogenic phenotypes induced by TFAP2A or NUTM2A‐AS1, while miR‐409‐5p restoration suppressed SLC35F2 expression and cell‐cycle progression. Our study supports a TFAP2A/NUTM2A‐AS1/miR‐409‐5p/SLC35F2 regulatory model that contributes to LUAD progression. These findings suggest that the TFAP2A/NUTM2A‐AS1/miR‐409‐5p/SLC35F2 axis contributes to LUAD progression and may provide potential biomarkers and therapeutic targets.

## 1 Introduction

Lung cancer (LC) remains the leading cause of cancer‐related morbidity and mortality globally [1]. Histologically, LC is classified into two primary subtypes: non‐small cell lung cancer (NSCLC) and small cell lung cancer (SCLC). NSCLC, comprising lung adenocarcinoma (LUAD) and lung squamous cell carcinoma (LUSC), accounts for approximately 85% of all LC cases, whereas SCLC accounts for 15% [2]. Within the NSCLC classification, LUAD represents the most prevalent subtype [3, 4, 5]. Despite advancements in early detection and precision therapies, the 5‐year overall survival rate for LUAD patients remains at approximately 19% [6, 7, 8]. Over the past decade, precision medicine strategies targeting specific driver genes have improved survival outcomes [9]. Consequently, elucidating the molecular mechanisms underlying LUAD progression is critical for identifying novel biomarkers and developing effective therapeutic strategies [10, 11].

While cancer research has historically focused on protein‐coding genes, non‐coding RNAs (ncRNAs), including microRNAs (miRNAs), small nuclear RNAs (snRNAs), PIWI‐interacting RNAs (piRNAs) and long non‐coding RNAs (LncRNAs), have recently emerged as key regulators in oncology. These molecules are implicated in various malignant processes, such as invasion, stemness and drug resistance [12]. LncRNAs are defined as transcripts lacking functional open reading frames (ORFs) that operate via diverse mechanisms, acting as signalling molecules, guides, decoys or scaffolds within competitive endogenous RNA (ceRNA) networks [13, 14]. The ceRNA hypothesis has gained prominence for its role in modulating gene expression and driving aggressive cancer phenotypes [15]. Thus, ncRNAs hold promise as both biomarkers and therapeutic targets in lung diseases [16]. LncRNA NUTM2A‐AS1 is a recently identified transcript that is dysregulated in multiple tumour types. For instance, NUTM2A‐AS1 levels are significantly elevated in NSCLC tissues compared to healthy controls [17]. Furthermore, its expression is upregulated in gastric cancer (GC) cells, where its suppression attenuates proliferation, invasion and drug resistance [18]. These findings suggest that LncRNA NUTM2A‐AS1 may serve as a potential biomarker for cancer detection and monitoring. However, the precise mechanisms governing NUTM2A‐AS1 function in LUAD progression remain to be fully elucidated.

The *Homo sapiens* solute carrier family 35 member F2 (SLC35F2) was first identified by Stankovic et al. in the context of ataxia‐telangiectasia syndrome [19]. Subsequent studies have investigated the expression and biological functions of SLC35F2 across various malignancies. Nyquist et al. demonstrated that SLC35F2 expression correlates with androgen levels in prostate cancer and is modulated by androgen receptor signalling [20]. Kotolloshi et al. reported that SLC35F2 acts as a critical promoter of bladder cancer (BC) cell growth, migration and invasion [21]. Additionally, He et al. implicated SLC35F2 in papillary thyroid carcinoma (PTC) carcinogenesis via the mitogen‐activated protein kinase (MAPK) pathway [22]. These findings suggest that SLC35F2 may serve as a viable biomarker for prognosis and therapeutic evaluation in malignancies, including LUAD. Nevertheless, the specific function, regulatory mechanism and prognostic value of SLC35F2 in LUAD require further investigation.

In this study, we investigated the functional role of LncRNA NUTM2A‐AS1 in LUAD. We demonstrated that TFAP2A and NUTM2A‐AS1 promote LUAD cell growth in vitro and enhance xenograft tumour growth in vivo. Furthermore, we identified that SLC35F2 expression is modulated by the LncRNA NUTM2A‐AS1/miR‐409‐5p axis. Specifically, TFAP2A‐mediated upregulation of LncRNA NUTM2A‐AS1 drives LUAD development as a ceRNA‐like regulator of miR‐409‐5p, thereby relieving the repression of SLC35F2. While TFAP2A‐regulated LncRNAs have been reported in breast [23] and bladder cancers [24], their role in LUAD has remained unexplored. To our knowledge, this is the first study to integrate the TFAP2A/NUTM2A‐AS1/miR‐409‐5p/SLC35F2 axis in the context of LUAD, addressing a significant gap in the current literature.

## 2 Materials and Methods

### 2.1 Bioinformatics Analysis

We analysed The Cancer Genome Atlas (TCGA) data via UALCAN (https://ualcan.path.uab.edu/index.html) to evaluate LncRNA and mRNA expression (|logFC| > 1.5, *p*adj < 0.05; cases: 533, controls: 59) and clinical data for LUAD (accessed December 2025). Potential binding sites between miR‐409‐5p and LncRNA NUTM2A‐AS1 were predicted using ENCORI/starBase (https://rnasysu.com/encori/). SLC35F2 expression data across cancers were obtained from the GEPIA dataset (http://gepia.cancer‐pku.cn/index.html).

### 2.2 Tissue Samples

A total of 34 paired LUAD and adjacent normal tissue (ANT) specimens were collected from patients undergoing surgery at Tongren Hospital, Affiliated to Shanghai Jiao Tong University School of Medicine. Following resection, tissue samples were immediately snap‐frozen in liquid nitrogen and stored at −80°C until analysis. Informed consent was obtained from all patients. The study was approved by the Institutional Ethics Committee of Tongren Hospital and all protocols adhered to the ethical guidelines established by the institution's research committee.

### 2.3 Animal Studies

Male BALB/c nude mice (4–6 weeks old, 18–20 g) were purchased from Shanghai SLAC Laboratory Animal Co. Ltd. and maintained in pathogen‐free conditions with ad libitum access to food and water. Fifty mice were randomly allocated into 10 groups (*n* = 5 per group) in a blinded manner. A549 cells (2 × 10^6^ cells per mouse) were injected subcutaneously. To investigate the role of TFAP2A, groups were assigned as follows: Group 1: OE‐NC; Group 2: OE‐TFAP2A; Group 3: sh‐NC; Group 4: sh‐TFAP2A‐1; Group 5: sh‐TFAP2A‐2. To study the role of LncRNA NUTM2A‐AS1, groups were assigned as follows: Group 1: OE‐NC; Group 2: LncRNA NUTM2A‐AS1 overexpression; Group 3: sh‐NC; Group 4: sh‐NUTM2A‐AS1‐1; Group 5: sh‐NUTM2A‐AS1‐2. All animal experiments complied with the Guidelines for the Care and Use of Laboratory Animals and were approved by the Institutional Animal Care and Use Committee of Tongren Hospital. Tumour volume was measured every other day. Thirty‐five days post implantation, mice were humanely euthanised using rapid cervical dislocation under anaesthesia, followed by tumour dissection and weight measurement. Tumour volume was calculated as V = length × width^2^/2.

### 2.4 Cell Culture

Immortalised normal bronchial epithelial cell line BEAS‐2B (CRL‐3588), human LUAD cell lines (A549 (Cat# CCL‐185) and H358 (Cat# CRL‐5807)) and an EGFR‐mutant LUAD cell line HCC827 (EGFR Exon 19 deletion, CRL‐2868) were obtained from ATCC (USA). Cells were cultured in T‐75 flasks with F‐12K (A549), RPMI‐1640 (H358 and HCC827), BEGM (BEAS‐2B) supplemented with 10% FBS (Cat# 10099‐141, Gibco, USA) at 37°C in a humidified 5% CO_2_ atmosphere. Fresh medium was replenished every 48 h. All cell lines were authenticated by STR profiling and tested negative for mycoplasma contamination.

### 2.5 Western Blot Analysis

Total protein was extracted using RIPA Lysis buffer (Cat# P0013B, Beyotime, China) and quantified via the BCA assay (Cat# P0012, Beyotime, China). Protein samples were separated on a 12% polyacrylamide gel (Cat# 4561043, Bio‐Rad, USA), transferred to 0.45 μm PVDF membranes (Cat# 10600023, Amersham, USA) and blocked with non‐fat milk. Membranes were incubated with primary antibodies overnight at 4°C, followed by secondary antibodies for 2 h at room temperature. Bands were visualised using the ChemiDoc MP Imaging System (Cat# 12003154, Bio‐Rad, USA) with chemiluminescent HRP substrate (Cat# WBKLS0500, Millipore, USA). GAPDH served as the loading control. Antibody details are provided in Table S1.

### 2.6 RNA Extraction and qRT‐PCR

Total RNA was extracted using Trizol reagent (Cat#15596026, Life Technologies, USA). cDNA synthesis was performed using the cDNA Synthesis Kit (Vazyme, USA) according to the manufacturer's instructions. qRT‐PCR was conducted using the SsoFast EvaGreen Supermix system (Cat#1725201, Bio‐Rad, USA). GAPDH was used as an internal control for mRNA and LncRNA, whereas U6 snRNA was used for miRNA normalisation. Primers are listed in Table S2.

### 2.7 Cell Transfection

LncRNA NUTM2A‐AS1 overexpression plasmid, its corresponding negative control (NC), pcDNA3.1‐TFAP2A overexpression plasmid and an empty pcDNA3.1 vector (OE‐NC) were obtained from RiboBio (China). Small interfering RNAs (siRNAs) targeting TFAP2A, LncRNA NUTM2A‐AS1 and SLC35F2, along with scrambled negative control siRNAs, were also synthesised by RiboBio (China). Transient transfections were performed in A549, H358 and HCC827 cells using Lipofectamine 2000 (Cat# 11668019, ThermoFisher, USA) according to the manufacturer's instructions. Cells were harvested 24–48 h post‐transfection for subsequent functional assays (e.g., cell viability, cell cycle, apoptosis) and expression analysis. All these transfection efficiency validation results are shown in Figure S1.

### 2.8 Stable Cell Line Generation (For Long‐Term Studies and In Vivo Experiments)

To generate stable cell lines with modulated expression of TFAP2A, LncRNA NUTM2A‐AS1 or SLC35F2, short hairpin RNAs (shRNAs) targeting human TFAP2A, NUTM2A‐AS1 or SLC35F2, as well as specific lentiviral overexpression constructs for TFAP2A and LncRNA NUTM2A‐AS1, were cloned into the hU6‐MCS‐CBh‐gcGFP‐IRES‐puromycin lentiviral vector (Genechem, China). HEK293T cells were used for lentivirus packaging. A549, H358 or HCC827 cells were then transduced with the filtered lentivirus. Stable cell lines were established through selection with 0.5 mg/L puromycin (Cat#P8833, Sigma‐Aldrich; Merck KGaA, USA) for 10 days, as previously described [25]. Scrambled shRNAs or empty lentiviral vectors were used as negative controls. All these transfection efficiency validation results are shown in Figure S1.

### 2.9 Cell Viability Assay

Cell viability was assessed using the CCK‐8 assay (Cat# CK04, Dojindo, Japan) [26]. A549, H358, or HCC827 cells were seeded in 96‐well plates (6 × 10^3^ cells/well) and incubated for 24 h prior to treatment. Following a 3‐h incubation with CCK‐8 reagent, absorbance at 450 nm was measured using a microplate spectrophotometer (EnVision, PerkinElmer, USA).

### 2.10 Cell Cycle Analysis

Cell cycle distribution was analysed 24 h post‐transfection using a cell cycle assessment kit (Cat# ab139418, Abcam, UK). Cells were fixed in 95% ethanol overnight and stained with 50 μg/mL propidium iodide (PI) in the dark for 30 min. Flow cytometry was used to quantify cell cycle phases.

### 2.11 EdU Incorporation Assay

Cell proliferation was assessed via 5‐ethynyl‐2′‐deoxyuridine (EdU) assay for newly synthesised DNA. Transfected/transduced A549 cells were seeded, cultured overnight and incubated with EdU for 2 h at 37°C. Cells were then washed, fixed with 4% paraformaldehyde and permeabilised with Triton X‐100. EdU signals were detected by click chemistry and nuclei were stained with DAPI. Fluorescence images were acquired with unified parameters. EdU‐positive ratio was quantified by counting positive nuclei against total stained nuclei in no fewer than five random fields per sample. All experiments were performed in triplicate at minimum.

### 2.12 Colony Formation Assay

Colony formation assay was used to evaluate long‐term clonogenic ability of LUAD cells. A549, H358 and HCC827 cells with gene overexpression, knockdown or rescue were seeded at 500–1000 cells per well and cultured for 14 days with regular medium renewal. Colonies were fixed with 4% paraformaldehyde or methanol, stained with crystal violet, then counted manually or via ImageJ. Colonies with over 50 cells were quantified and normalised to controls. All assays were conducted in triplicate and repeated at least three times.

### 2.13 Nuclear/Cytoplasmic Fractionation

Nuclear‐cytoplasmic fractionation was conducted to analyse the subcellular distribution of NUTM2A‐AS1 and miR‐409‐5p in A549, H358 and HCC827 cells. Cytoplasmic and nuclear RNAs were separately extracted and quantified by qRT‐PCR. GAPDH and U6 served as cytoplasmic and nuclear markers, respectively. RNA distribution was calculated based on relative expression in each fraction. All experiments were repeated in three independent biological replicates.

### 2.14 Apoptosis Analysis

Cell apoptosis was evaluated using the Annexin V‐FITC/PI Apoptosis Detection Kit (Cat# C1062S, Beyotime, China) according to the manufacturer's protocol. Briefly, 48 h post‐transfection, HCC827 cells were harvested, washed with cold PBS and resuspended in 195 μL of Annexin V binding buffer. Cells were then incubated with 5 μL Annexin V‐FITC and 10 μL PI for 15 min at room temperature in the dark. The apoptotic rate was quantified using a flow cytometer (BD Biosciences, USA).

### 2.15 Transwell Migration and Invasion Assays

Cell migration and invasion were assessed using 24‐well Transwell chambers (8‐μm pore size; Corning, USA). For the invasion assay, the upper chambers were pre‐coated with Matrigel (BD Biosciences, USA). Approximately 5 × 10^4^ HCC827 cells suspended in 200 μL serum‐free RPMI‐1640 were seeded into the upper chamber, while 600 μL RPMI‐1640 containing 10% FBS was added to the lower chamber as a chemoattractant. After 24 h (for migration) or 48 h (for invasion) of incubation, cells were fixed with 4% paraformaldehyde, stained with 0.1% crystal violet and counted in five random microscopic fields.

### 2.16 Chromatin Immunoprecipitation (ChIP)

ChIP assays were performed using the Millipore EZ Magna ChIP A/G kit (Cat# 17‐10085, Millipore, USA). A549, H358 or HCC827 cells were cross‐linked with 1% formaldehyde at 25°C for 10 min. Lysates were incubated overnight with anti‐TFAP2A antibody (Abcam, USA) or IgG control, along with protein A/G agarose beads. Enriched DNA was quantified via qRT‐PCR.

### 2.17 RNA Immunoprecipitation Assay

RNA immunoprecipitation (RIP) was used to verify whether LncRNA NUTM2A‐AS1, miR‐409‐5p and SLC35F2 mRNA coexisted in the RNA‐induced silencing complex. The experiment was carried out in A549, H358 and HCC827 cells using the Magna RIP Kit following standard protocols with slight adjustments. Briefly, cell lysates were incubated with magnetic beads coupled to anti‐AGO2 antibody or control IgG. After washing and proteinase K digestion, coprecipitated RNAs were purified and reverse‐transcribed. RIP enrichment was calculated as percentage of input and normalised to IgG control. All assays were repeated in triplicate. The AGO2 enrichment of these RNAs indicated their involvement in the AGO2‐related miRNA regulatory complex.

### 2.18 Microarray Analysis

Total RNA was extracted from paired LUAD and adjacent normal tissues using TRIzol reagent and purified with the RNeasy Mini Kit. RNA quantity and integrity were assessed using a NanoDrop spectrophotometer and an Agilent 2100 Bioanalyzer. For LncRNA microarray analysis, biotin‐labelled cRNA was generated using the GeneChip 3′ IVT PLUS Reagent Kit (Affymetrix/Thermo Fisher Scientific, USA) according to the manufacturer's instructions. Fragmented labelled cRNA was hybridised to Affymetrix human expression arrays, followed by washing, staining and scanning using the GeneChip system. For miRNA microarray analysis, total RNA was labelled using the FlashTag Biotin HSR RNA Labeling Kit (Affymetrix/Thermo Fisher Scientific, USA) according to the manufacturer's protocol and hybridised to Affymetrix GeneChip miRNA arrays. Differential miRNA expression analysis was performed as previously described [27].

### 2.19 Dual‐Luciferase Reporter Assay

Promoter fragments of the NUTM2A‐AS1 gene (350, 500, 1000 and 6000 bp) were cloned into the pGL3 vector. A549 cells were co‐transfected with reporter plasmids (1 μg) and Renilla luciferase vectors (1 μg) using Lipofectamine 2000. Luciferase activity was measured 48 h later using the Dual‐Luciferase Reporter Assay System (Cat# E1910, Promega, USA).

### 2.20 Biotinylated miRNA Pull‐Down Assay

Biotin‐labelled miR‐409‐5p mimics or NC mimics were transfected into A549, H358 or HCC827 cells (1 × 10^6^ cells, 100 pmol). After 48 h, cells were lysed in buffer containing RNase inhibitors and protease inhibitors. Lysates were incubated with streptavidin‐coated beads to isolate biotin‐miRNA complexes as described previously [28]. Co‐precipitated LncRNA NUTM2A‐AS1 or SLC35F2 levels were quantified by qRT‐PCR.

### 2.21 Statistical Analysis

Statistical analyses were performed using GraphPad Prism 9.0. All in vitro experiments were performed with three independent biological replicates, each with technical triplicates where applicable. Data are presented as the mean ± SD unless otherwise indicated. For paired comparisons between LUAD tissues and matched adjacent normal tissues, paired Student's *t*‐test or Wilcoxon matched‐pairs signed‐rank test was used according to data distribution. For comparisons between two independent groups, unpaired two‐tailed Student's *t*‐test or Mann–Whitney *U* test was applied. For multiple‐group comparisons, one‐way ANOVA followed by Tukey's or Dunnett's post hoc test was used as appropriate. CCK‐8 assays measured at multiple time points and xenograft tumour growth curves were analysed using two‐way ANOVA or repeated‐measures two‐way ANOVA followed by multiple‐comparison correction. Kaplan–Meier survival curves were compared using the log‐rank test. Correlations between gene expression levels were analysed using Pearson's or Spearman's correlation analysis depending on data distribution. Normality was assessed using Shapiro–Wilk test. Public database analyses were conducted according to the statistical methods implemented in UALCAN or GEPIA and adjusted *p*‐values were used where applicable. A two‐sided *p* < 0.05 was considered statistically significant.

## 3 Results

### 3.1 LncRNA NUTM2A‐AS1 Is Upregulated in LUAD Tissues

**FIGURE 1:**
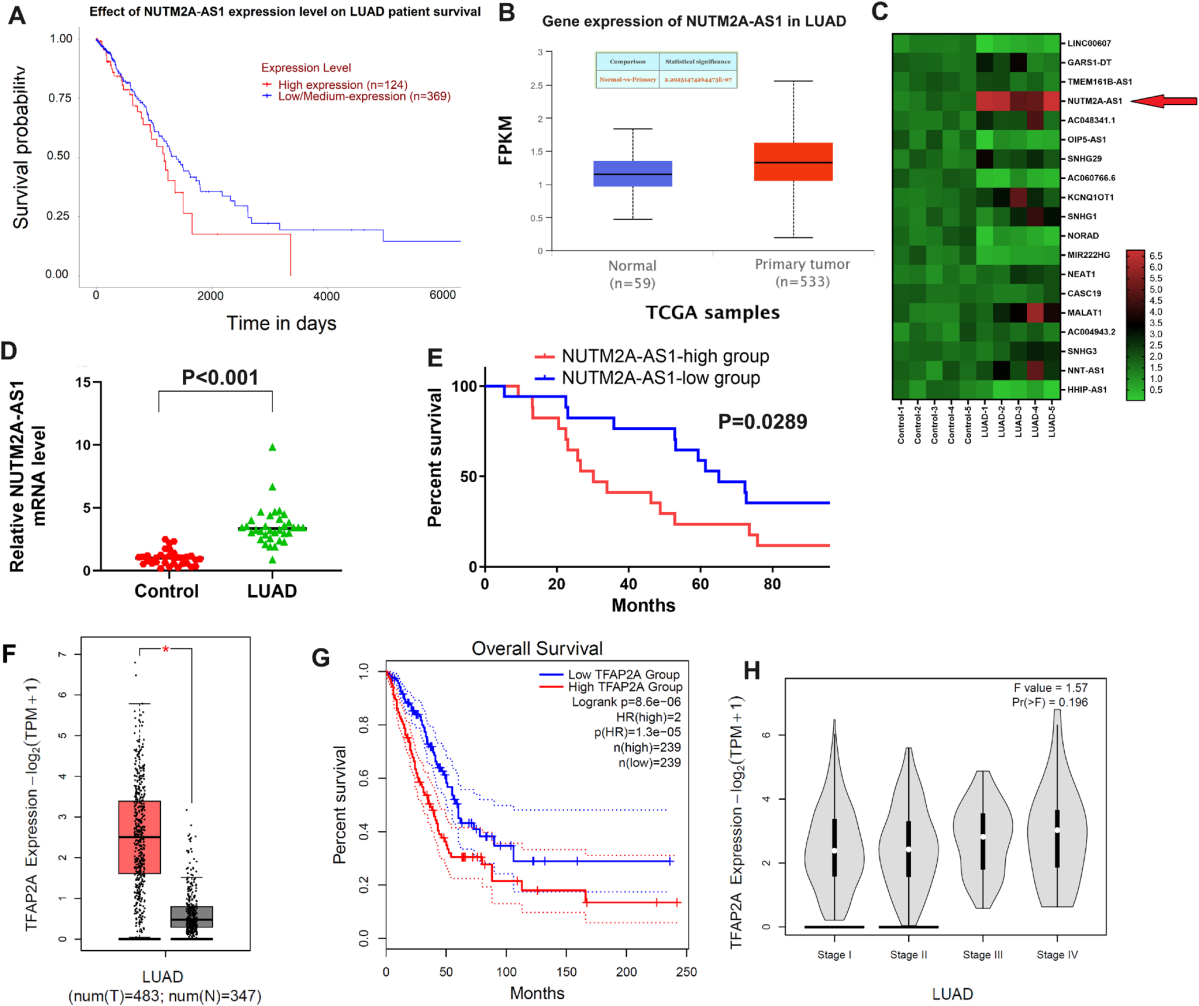
LncRNA NUTM2A‐AS1 is upregulated in LUAD tissues. (A) Kaplan–Meier survival analysis of LncRNA NUTM2A‐AS1 expression (high vs. low) from the UALCAN database. (B) Expression levels of LncRNA NUTM2A‐AS1 in LUAD cases (*n* = 533) versus controls (*n* = 59) from UALCAN. (C) Microarray analysis of LncRNA expression in paired LUAD cases and controls (*n* = 5). (D) qRT‐PCR validation of LncRNA NUTM2A‐AS1 expression in adjacent normal tissues (Control) and LUAD tissues (*n* = 34). (E) Mortality rates associated with low versus high LncRNA NUTM2A‐AS1 expression in the clinical cohort. (F) TFAP2A expression in LUAD cases (*n* = 483) versus controls (*n* = 347) from GEPIA. (G) Prognostic value of TFAP2A expression in LUAD patients. (H) TFAP2A expression across different clinical stages (Stage I–IV). *p < 0.05.

We analysed TCGA data via UALCAN. The results indicated that high expression of LncRNA NUTM2A‐AS1 is significantly associated with poor prognosis in LUAD patients (Figure 1A). Notably, LncRNA NUTM2A‐AS1 expression was markedly elevated in LUAD tissues (*n* = 533) compared to normal controls (*n* = 59) (Figure 1B). We validated this upregulation in an independent cohort of 34 paired samples using qRT‐PCR and five samples via microarray (Figure 1C,D). In our limited clinical cohort, higher NUTM2A‐AS1 expression showed a trend poorer outcome (Figure 1E). Furthermore, subcellular fractionation revealed that NUTM2A‐AS1 is predominantly localised in the cytoplasm, similar to the cytoplasmic marker GAPDH, whereas U6 was restricted to the nucleus (Figure S3A–C). This cytoplasmic localisation supports the hypothesis that NUTM2A‐AS1 may function via a ceRNA mechanism. Collectively, these data confirm the upregulation and clinical significance of LncRNA NUTM2A‐AS1 in LUAD.

### 3.2 TFAP2A Is Highly Expressed in LUAD and Correlates With NUTM2A‐AS1

**FIGURE 2:**
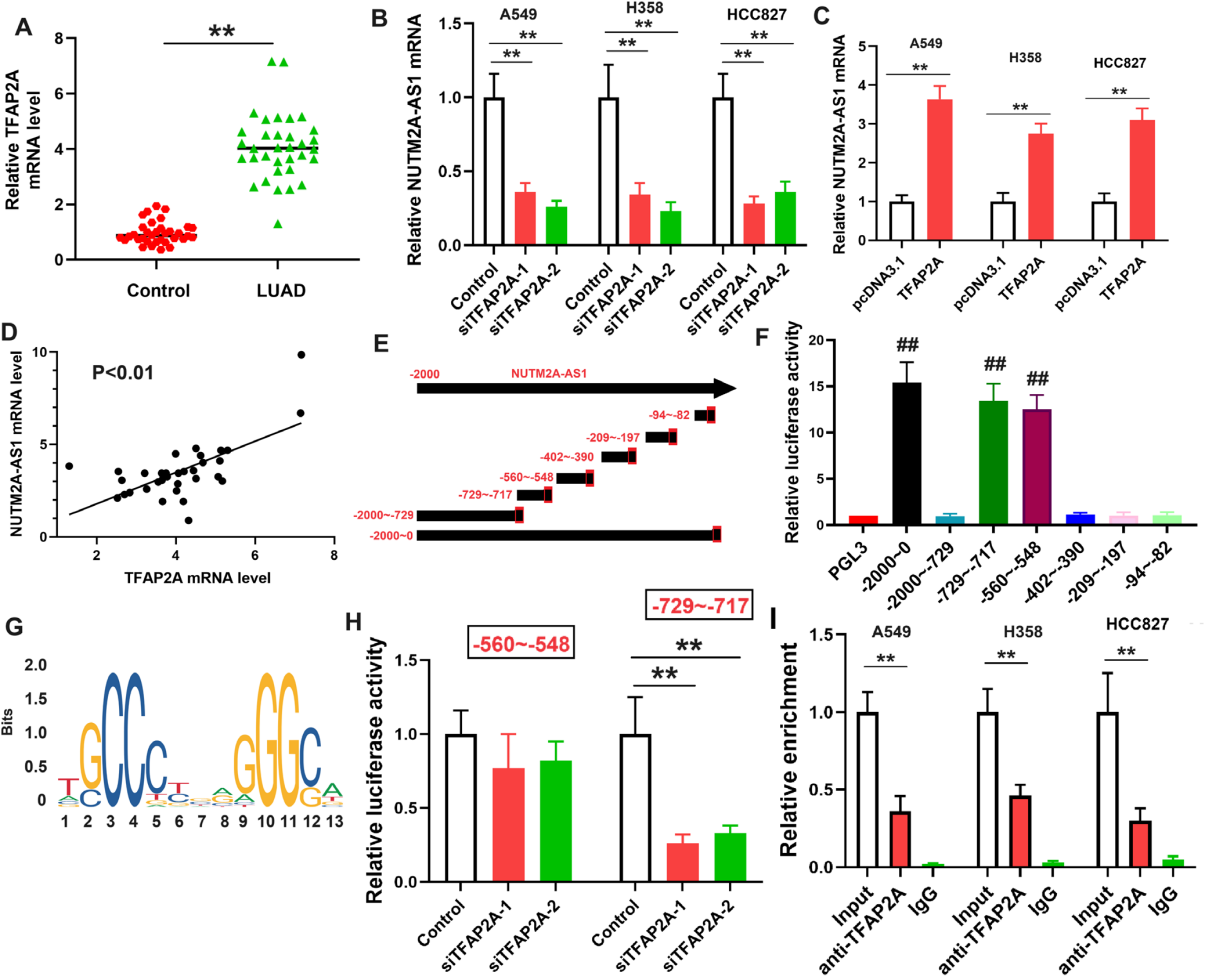
TFAP2A is upregulated in LUAD and transcriptionally regulates LncRNA NUTM2A‐AS1. (A) TFAP2A expression in adjacent normal tissues (Control) and LUAD tissues (*n* = 34). (B, C) LncRNA NUTM2A‐AS1 expression in A549, H358 and HCC827 cells following TFAP2A overexpression or knockdown. GAPDH was used for normalisation. (D) Linear correlation between TFAP2A and LncRNA NUTM2A‐AS1 expression in LUAD tissues (*n* = 34). (E) Schematic of LncRNA NUTM2A‐AS1 promoter deletion constructs cloned into the pGL3‐basic vector. (F) Luciferase activity of promoter deletion mutants in A549 cells. (G) Predicted TFAP2A binding sites on the LncRNA NUTM2A‐AS1 promoter. (H) Luciferase activity of specific deletion mutants in TFAP2A‐knockdown A549 cells. (I) ChIP‐qPCR analysis of TFAP2A binding to the LncRNA NUTM2A‐AS1 promoter in A549, H358 and HCC827 cells. Genomic DNA input was 1%. Data represent mean ± SD of three independent experiments. **p < 0.01.

Analysis of the GEPIA dataset (347 controls, 483 LUAD cases) revealed that TFAP2A is significantly upregulated in LUAD tissues (Figure 1F). High TFAP2A expression was also associated with poor overall survival (Figure 1G) and advanced clinical stages (Stage III/IV vs. Stage I/II) (Figure 1H). qRT‐PCR analysis of our 34 paired samples confirmed elevated TFAP2A levels in LUAD tissues (Figure 2A), consistent with previous reports [29, 30]. The mRNA and protein levels of TFAP2A in normal lung epithelial cells (BEAS‐2B) and in LUAD cell lines (A549, H358 and HCC827 cells) were measured by qRT‐PCR and Western blotting. The results showed a significant increase of mRNA and protein levels of TFAP2A in LUAD cell lines compared to normal lung epithelial cells (Figure S2). Given the concurrent upregulation of NUTM2A‐AS1 and TFAP2A, we investigated their potential regulatory relationship. In A549, H358 and HCC827 cells, TFAP2A knockdown significantly reduced NUTM2A‐AS1 levels, whereas TFAP2A overexpression increased them (Figure 2B,C). Correlation analysis in the 34 clinical samples demonstrated a moderate positive correlation between TFAP2A and NUTM2A‐AS1 mRNA levels (*p* < 0.01) (Figure 2D). These findings suggest that TFAP2A modulates LncRNA NUTM2A‐AS1 expression during LUAD progression.

### 3.3 TFAP2A Transcriptionally Activates LncRNA NUTM2A‐AS1

To elucidate the mechanism driving NUTM2A‐AS1 upregulation, we analysed its promoter region (retrieved from UCSC Genome Browser). Luciferase reporter assays using truncated promoter constructs in A549 cells (Figure 2E) identified the −729 to −717 bp region as crucial for transcriptional activity (Figure 2F). JASPAR database analysis predicted potential TFAP2A binding sites within this region (Figure 2G). To confirm NUTM2A‐AS1 as a direct transcriptional target of TFAP2A, we measured the luciferase activity of deletion mutants (pGL3‐del‐717/729 and pGL3‐del‐548/560) in TFAP2A‐knockdown A549 cells. Deletion of the −729~−717 nt region significantly attenuated luciferase activity (Figure 2H). Furthermore, ChIP assays demonstrated direct binding of TFAP2A to the NUTM2A‐AS1 promoter (−729~−717 nt) in A549, H358 and HCC827 cells (Figure 2I). These results confirm that TFAP2A directly regulates LncRNA NUTM2A‐AS1 transcription in LUAD.

### 3.4 TFAP2A Promotes LUAD Progression In Vitro and In Vivo

**FIGURE 3:**
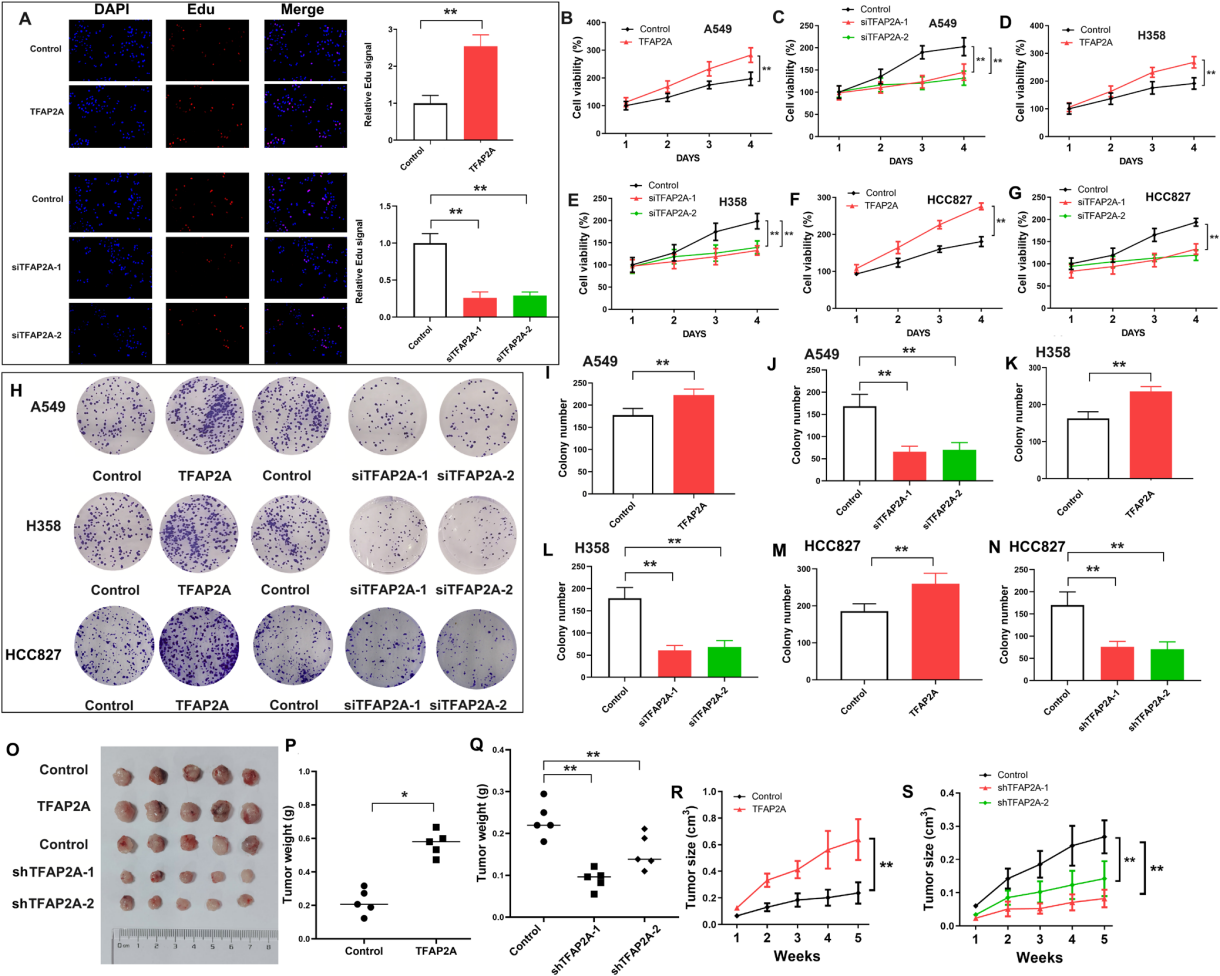
TFAP2A promotes LUAD progression in vitro and in vivo. (A) EdU proliferation assay in A549 cells with TFAP2A overexpression or knockdown. (B–G) CCK‐8 viability assays in A549, H358 and HCC827 cells following TFAP2A modulation. (H–N) Colony formation assays in A549, H358 and HCC827 cells. (O–S) Tumour volume and weight of xenografts derived from TFAP2A‐overexpressing or knockdown A549 cells. Data represent mean ± SD of three independent experiments. **p < 0.01.

We next evaluated the functional impact of TFAP2A. EdU assays showed that TFAP2A overexpression enhanced cell proliferation, while its knockdown suppressed it (Figure 3A). Consistent results were obtained from CCK‐8 assays (Figure 3B–G). Colony formation assays further indicated that TFAP2A overexpression increased clonogenicity, whereas knockdown resulted in a 50%–70% reduction in A549, H358 and HCC827 cells (Figure 3H–N). To assess tumorigenicity in vivo, we established xenograft models using A549 cells with stable TFAP2A knockdown or overexpression. TFAP2A overexpression significantly increased tumour volume and weight, while TFAP2A knockdown exerted the opposite effect (Figure 3O–S). These findings indicate that TFAP2A acts as an oncogenic driver in LUAD.

### 3.5 LncRNA NUTM2A‐AS1 Promotes LUAD Progression In Vitro and In Vivo

**FIGURE 4:**
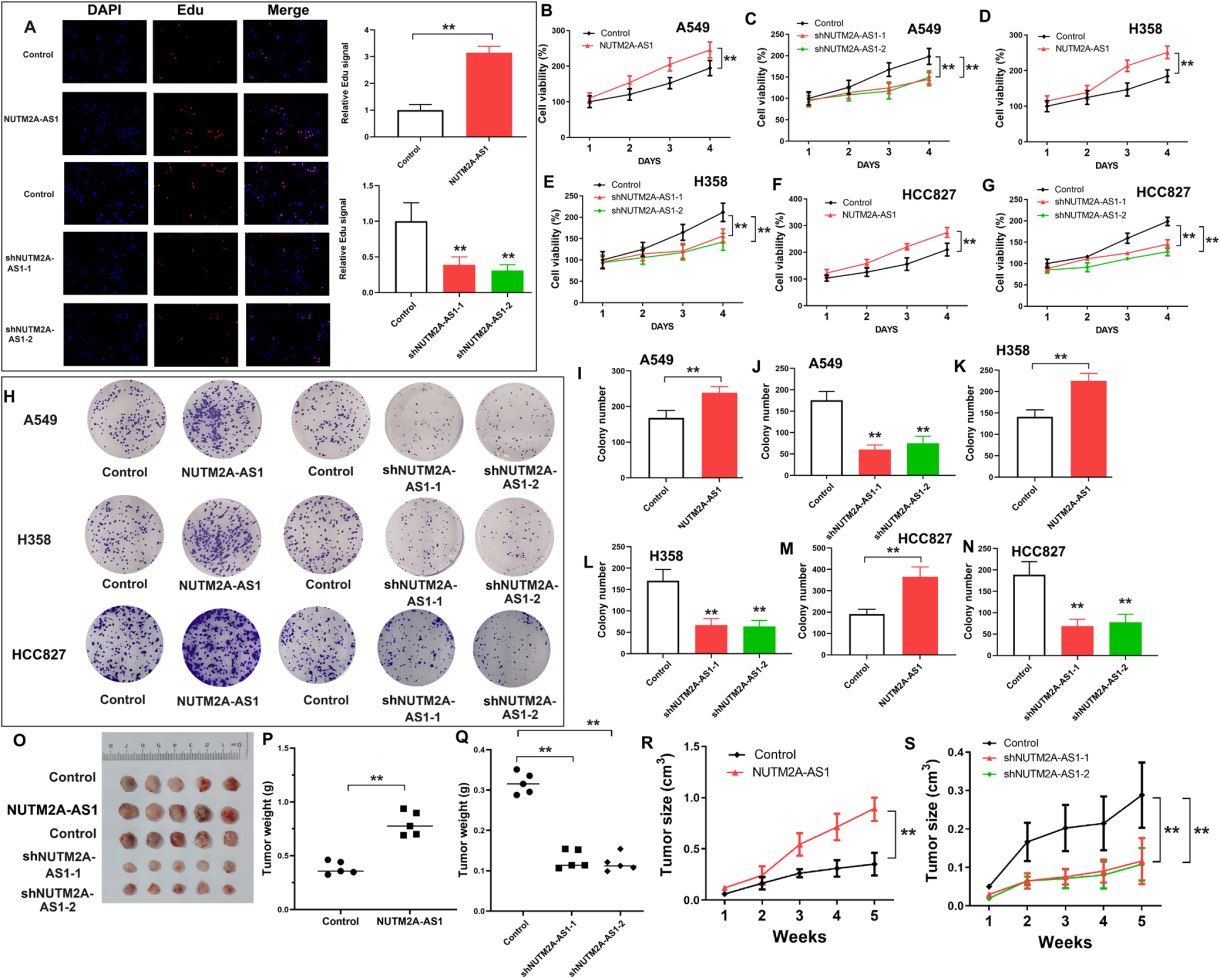
LncRNA NUTM2A‐AS1 promotes LUAD progression in vitro and in vivo. (A) EdU proliferation assay in A549 cells with LncRNA NUTM2A‐AS1 overexpression or knockdown. (B–G) CCK‐8 viability assays in A549, H358 and HCC827 cells. (H–N) Colony formation assays in A549, H358 and HCC827 cells. (O–S) Tumour volume and weight of xenografts derived from LncRNA NUTM2A‐AS1‐modulated A549 cells. Data represent mean ± SD of three independent experiments. **p < 0.01.

EdU and CCK‐8 assays revealed that NUTM2A‐AS1 overexpression significantly enhanced cell proliferation and viability, whereas its knockdown suppressed these phenotypes in A549, H358 and HCC827 cells (Figure 4A–G). Colony formation assays corroborated these findings, showing a 60%–80% decrease in clonogenicity upon NUTM2A‐AS1 knockdown (Figure 4H–N). In vivo xenograft experiments confirmed that NUTM2A‐AS1 overexpression promotes tumour growth, while its silencing significantly inhibits tumour size and weight (Figure 4O–S). These data support the role of LncRNA NUTM2A‐AS1 as a promoter of LUAD progression.

### 3.6 LncRNA NUTM2A‐AS1 Regulates SLC35F2 Expression to Drive LUAD

**FIGURE 5:**
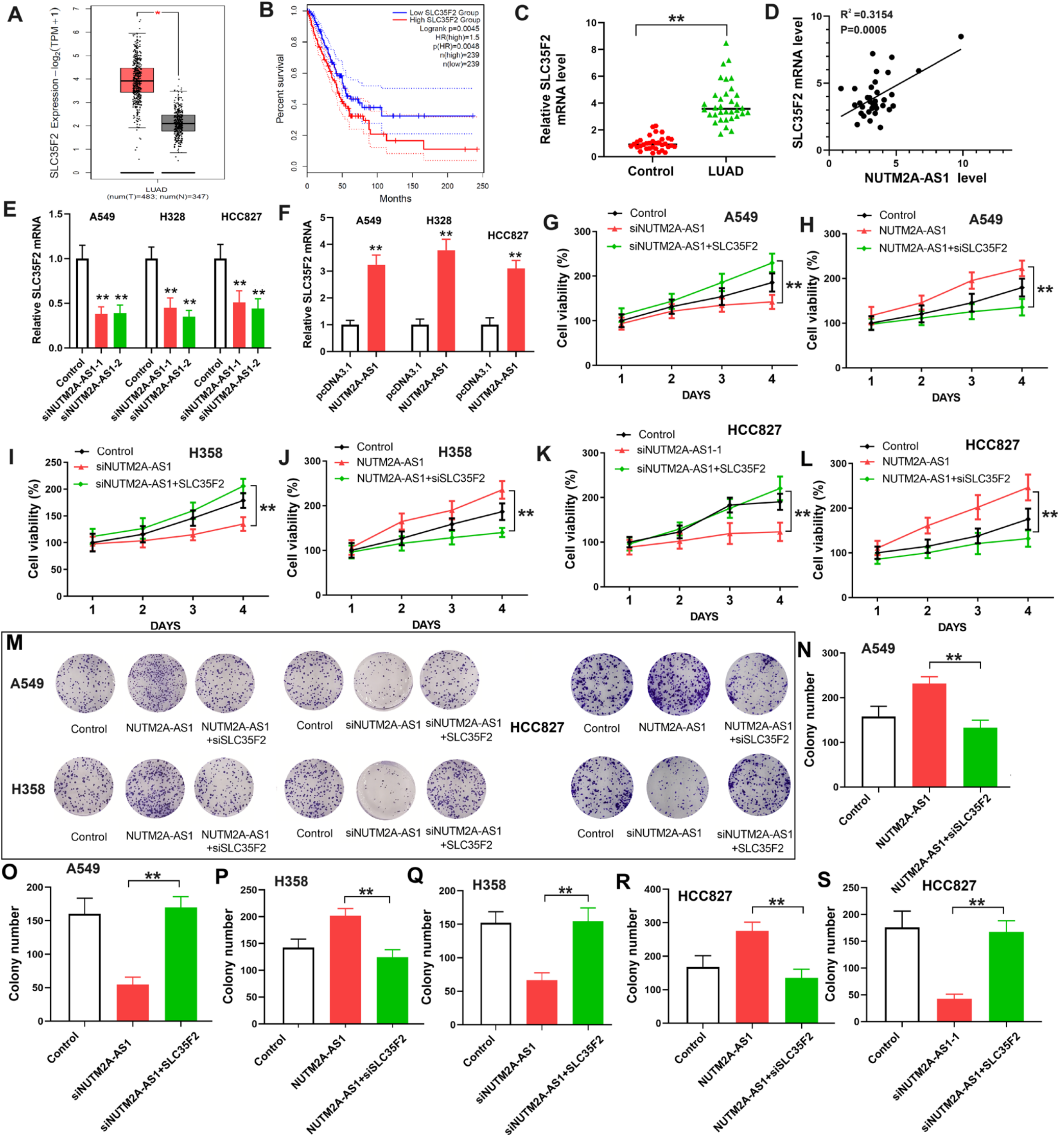
LncRNA NUTM2A‐AS1 regulates SLC35F2 expression to promote LUAD progression. (A) SLC35F2 expression in LUAD cases (*n* = 483) versus controls (*n* = 347) from GEPIA. (B) Prognostic significance of SLC35F2 expression. (C) SLC35F2 expression in adjacent normal tissues (Control) and LUAD tissues (*n* = 34). (D) Correlation between SLC35F2 and LncRNA NUTM2A‐AS1 expression in clinical samples. (E, F) SLC35F2 expression in A549, H358 and HCC827 cells following LncRNA NUTM2A‐AS1 modulation. (G–L) CCK‐8 viability assays showing rescue effects of SLC35F2 in A549, H358 and HCC827 cells. (M–S) Colony formation assays showing rescue effects of SLC35F2 in A549, H358 and HCC827 cells. Data represent mean ± SD of three independent experiments. **p < 0.01.

Recent studies implicate SLC35F2 in various cancers [31, 32]. GEPIA data analysis showed that SLC35F2 is upregulated in LUAD (Figure 5A) and associated with poor prognosis (Figure 5B). We confirmed high SLC35F2 expression in our LUAD cohort, which positively correlated with NUTM2A‐AS1 levels (*p* = 0.0005) (Figure 5C,D). Mechanistically, NUTM2A‐AS1 knockdown reduced SLC35F2 expression, while overexpression increased it (Figure 5E,F). Rescue experiments demonstrated that SLC35F2 upregulation restored cell viability and colony formation in NUTM2A‐AS1‐knockdown cells (Figure 5G–S). Conversely, SLC35F2 knockdown reversed the proliferative effects of NUTM2A‐AS1 overexpression (Figure 5G–S). These results suggest that NUTM2A‐AS1 promotes LUAD cell viability and proliferation by regulating SLC35F2. The mRNA and protein levels of SLC35F2 in normal lung epithelial cells (BEAS‐2B) and in LUAD cell lines (A549, H358 and HCC827 cells) were measured by qRT‐PCR and Western blotting. The results showed a significant increase of mRNA and protein levels of SLC35F2 in LUAD cell lines compared to normal lung epithelial cells (Figure S2).

### 3.7 NUTM2A‐AS1 Interacts With miR‐409‐5p and Regulates SLC35F2 Expression

**FIGURE 6:**
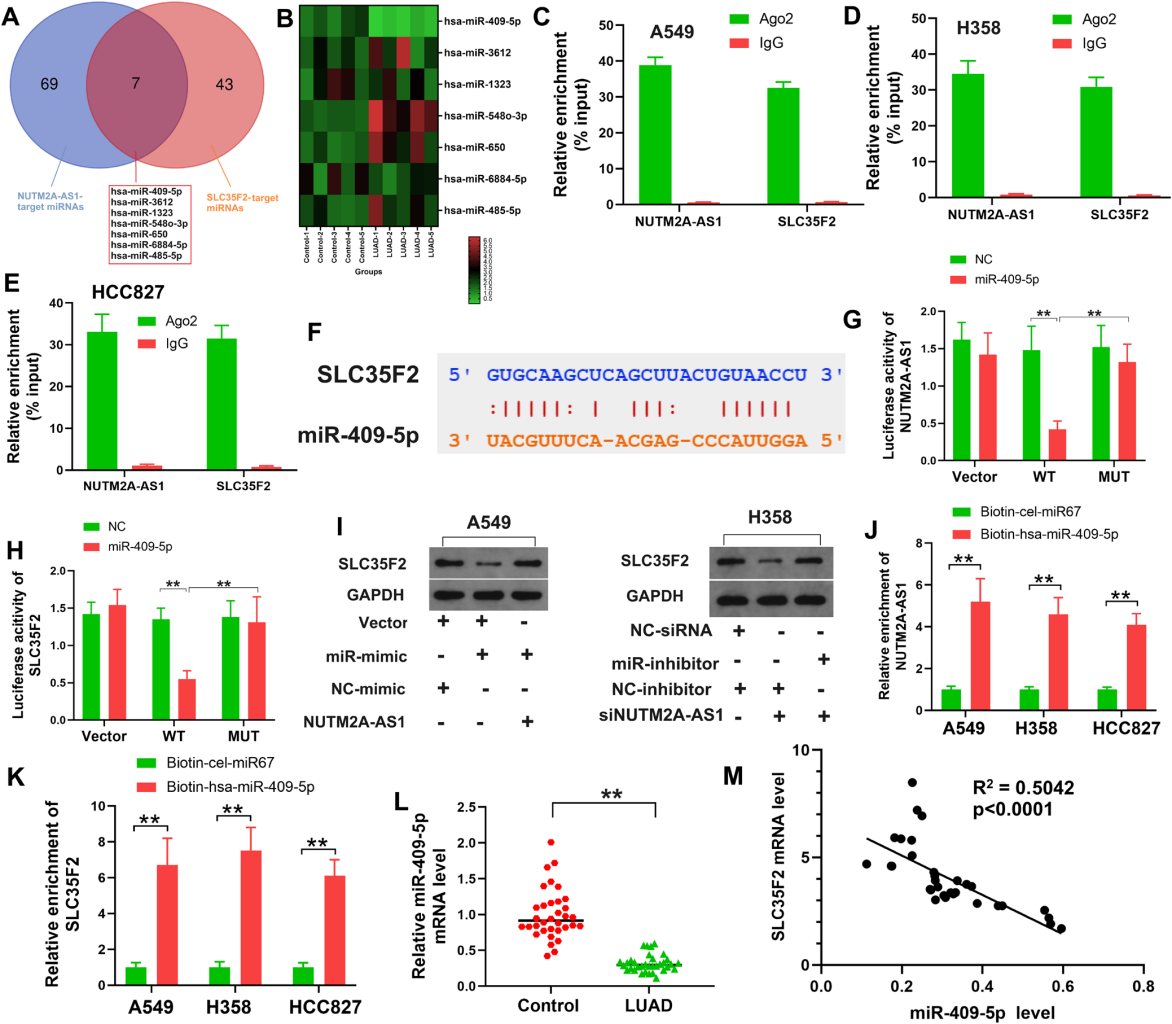
LncRNA NUTM2A‐AS1 regulates SLC35F2 via a miR‐409‐5p‐dependent mechanism. (A) Venn diagram of predicted miRNAs binding both SLC35F2 and LncRNA NUTM2A‐AS1 (ENCORI/starBase). (B) Microarray analysis of candidate miRNAs in LUAD tissues. (C–E) RIP assay showing enrichment of LncRNA NUTM2A‐AS1 and SLC35F2 in AGO2 immunoprecipitates in A549, H358 and HCC827 cells. (F) Predicted binding site between miR‐409‐5p and SLC35F2. (G, H) Dual‐luciferase reporter assays validating miR‐409‐5p binding to LncRNA NUTM2A‐AS1 and SLC35F2. (I) Western blot of SLC35F2 protein levels following modulation of LncRNA NUTM2A‐AS1 and miR‐409‐5p. (J, K) Biotinylated miR‐409‐5p pull‐down assay in A549, H358 and HCC827 cells. (L) miR‐409‐5p expression in adjacent normal tissues (Control) and LUAD tissues (*n* = 34). (M) Correlation between SLC35F2 and miR‐409‐5p expression in clinical samples. Data represent mean ± SD of three independent experiments. **p < 0.01.

Given the cytoplasmic localisation of NUTM2A‐AS1, we explored its potential ceRNA function. ENCORI analysis predicted 7 miRNAs with binding sites for both NUTM2A‐AS1 and SLC35F2 (Figure 6A). RIP assays confirmed the enrichment of both NUTM2A‐AS1 and miR‐409‐5p in AGO2 immunoprecipitates (Figure S3D–F). Microarray analysis of 5 LUAD tissues identified miR‐409‐5p as significantly downregulated (Figure 6B). RIP assays confirmed the enrichment of both NUTM2A‐AS1 and SLC35F2 mRNA in AGO2 immunoprecipitates (Figure 6C–E). The predicted binding site between miR‐409‐5p and SLC35F2 is shown in Figure 6F. Luciferase reporter assays validated the direct interaction of the predicted miR‐409‐5p‐binding region within NUTM2A‐AS1 and the 3′UTR of SLC35F2 (Figure 6G,H). Western blot analysis showed that miR‐409‐5p mimics suppressed SLC35F2 protein levels, while inhibitors increased them. Importantly, NUTM2A‐AS1 overexpression counteracted the suppressive effect of miR‐409‐5p mimics on SLC35F2 (Figure 6I). Biotinylated miRNA pull‐down assays further confirmed the direct physical interaction of miR‐409‐5p with both NUTM2A‐AS1 and SLC35F2 (Figure 6J,K). Clinically, miR‐409‐5p levels were significantly decreased in LUAD tissues (Figure 6L) and negatively correlated with SLC35F2 expression (*R* ^2^ = 0.5042, *p* < 0.0001; Figure 6M), supporting the existence of this regulatory axis in patients.

In normal lung epithelial BEAS‐2B cells, miR‐409‐5p was much higher than that in LUAD cells (A549, H358 and HCC827 cells), whereas NUTM2A‐AS1 level was significantly lower than that in LUAD cells (A549, H358 and HCC827 cells) (Figure S3G,H). Furthermore, subcellular fractionation confirmed that > 80% of both NUTM2A‐AS1 and miR‐409‐5p transcripts co‐localised within the cytoplasmic compartment (Figure S3A–C).

### 3.8 The NUTM2A‐AS1/miR‐409‐5p/SLC35F2 Axis Regulates Cell Proliferation

**FIGURE 7:**
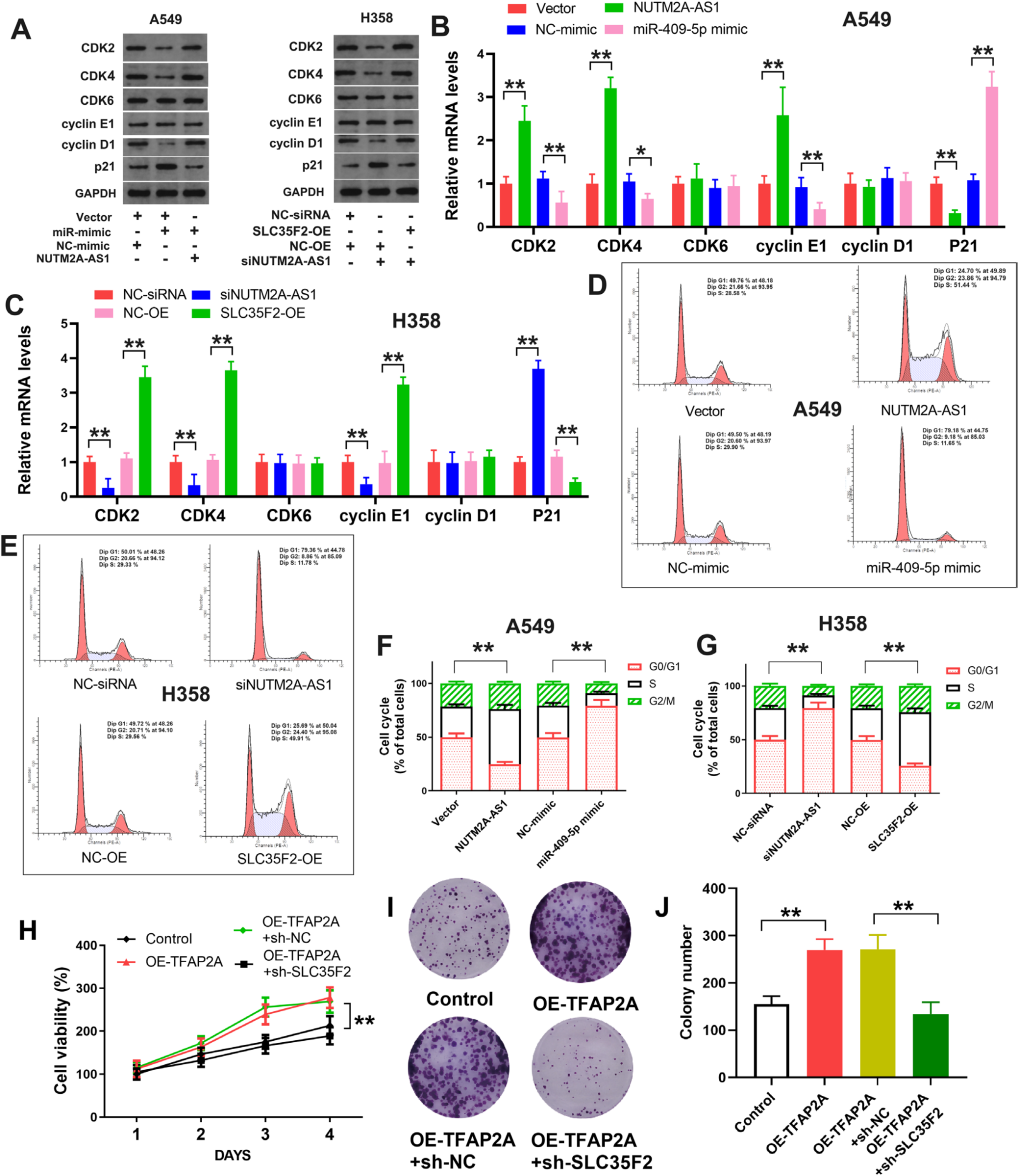
LncRNA NUTM2A‐AS1 modulates the cell proliferation by acting as a ceRNA‐like regulator of miR‐409‐5p to upregulate SLC35F2. (A–C) Western blot analysis of cell cycle markers in A549 and H358 cells. (D–G) Flow cytometry analysis of cell cycle distribution. (H–J) Rescue experiments using CCK‐8 and colony formation assays demonstrating that SLC35F2 knockdown attenuates TFAP2A‐induced cell viability and clonogenicity. Data represent mean ± SD of three independent experiments. **p < 0.01.

Finally, we examined the impact of this axis on the cell cycle. Western blot analysis of cell cycle markers in A549 and H358 cells and flow cytometry revealed that miR‐409‐5p mimics induced G0/G1 arrest and reduced the S‐phase fraction. This arrest was rescued by NUTM2A‐AS1 overexpression (Figure 7A–G). Similarly, SLC35F2 overexpression restored cell cycle progression in the presence of miR‐409‐5p mimics (Figure 7A–G). To confirm SLC35F2 as a downstream effector of the TFAP2A/NUTM2A‐AS1 axis, rescue experiments showed that SLC35F2 knockdown significantly attenuated the enhanced cell viability and colony formation induced by TFAP2A overexpression (Figure 7H–K).

### 3.9 The TFAP2A/NUTM2A‐AS1/SLC35F2 Axis Regulates Migration, Invasion, EMT‐Associated Changes and Apoptosis in LUAD Cells

**FIGURE 8:**
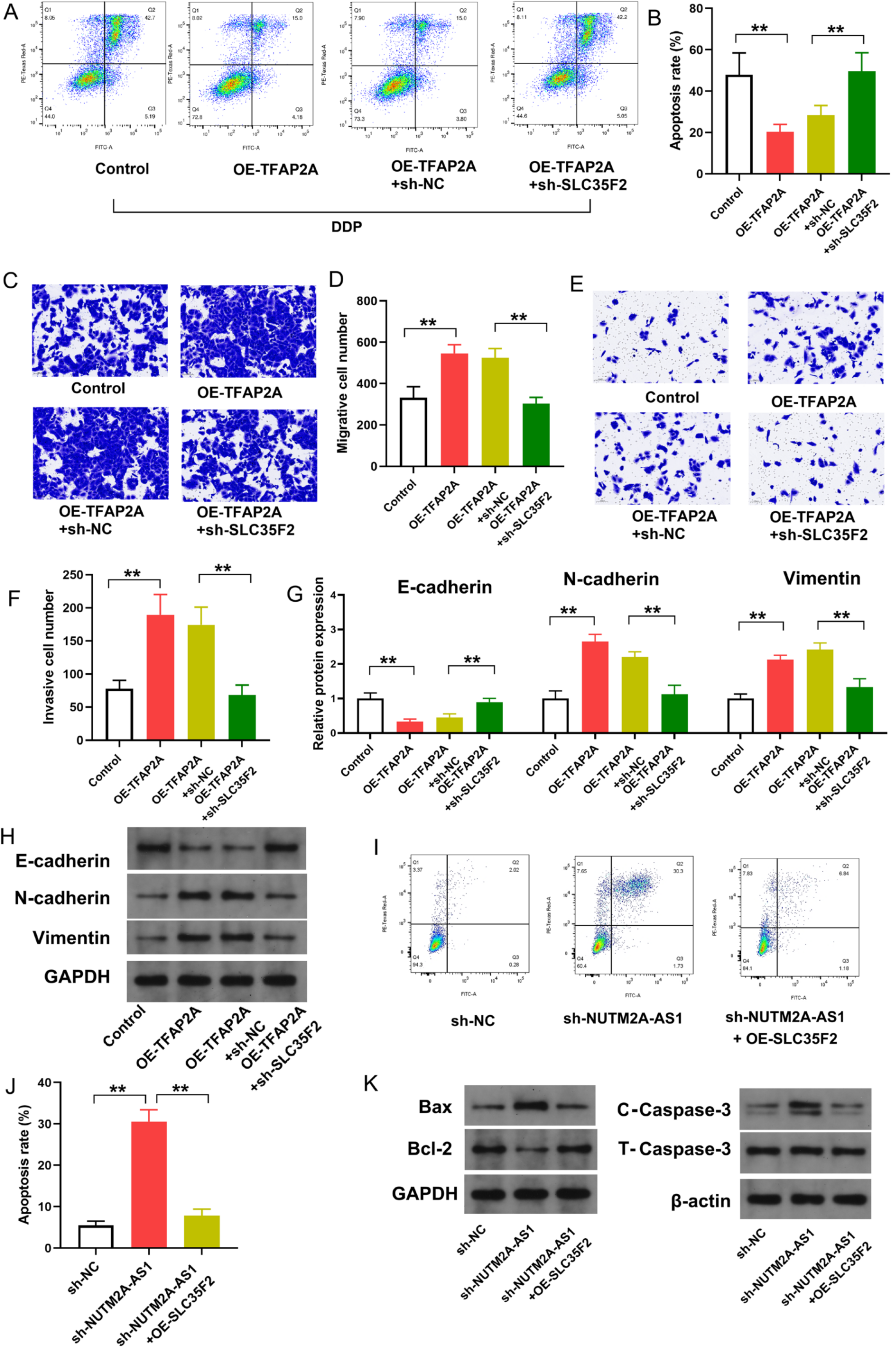
TFAP2A promotes LUAD cell survival, migration, invasion and EMT‐associated changes in an SLC35F2‐dependent manner in HCC827 cells. (A, B) Flow cytometry analysis of apoptosis in LUAD cells co‐transfected with TFAP2A overexpression plasmid (OE‐TFAP2A) and/or SLC35F2 shRNA (sh‐SLC35F2) in HCC827 cells. (C–F) Representative images and quantitative analysis of Transwell migration and invasion assays in HCC827 cells. TFAP2A overexpression remarkably enhances the migratory and invasive capacities of LUAD cells, which are effectively reversed by SLC35F2 silencing. (G, H) Western blot analysis of epithelial‐mesenchymal transition (EMT) markers in HCC827 cells. GAPDH was used as the loading control. (I, J) Flow cytometry analysis of apoptosis in HCC827 cells transfected with shRNA against NUTM2A‐AS1 (sh‐NUTM2A‐AS1) alone or co‐transfected with SLC35F2 overexpression plasmid (OE‐SLC35F2). (K) Western blot results of apoptosis markers in HCC827 cells. Data are presented as mean ± SD of three independent experiments. *p < 0.05, **p < 0.01.

To investigate whether the TFAP2A‐driven malignant phenotypes are dependent on downstream SLC35F2, we first performed rescue experiments focusing on cell motility. HCC827 cells were co‐transfected with TFAP2A overexpression plasmids (OE‐TFAP2A) and SLC35F2 shRNA (sh‐SLC35F2). Treatments of sh‐NC alone showed no significant impact on cell apoptosis (Figure S2D). Flow cytometric analysis showed that TFAP2A overexpression reduced apoptosis, whereas SLC35F2 knockdown restored apoptotic levels, indicating that SLC35F2 is required for TFAP2A‐mediated apoptosis evasion (Figure 8A,B). As shown in Figure 8C–H, overexpression of TFAP2A significantly enhanced cell migration and invasion and promoted the epithelial‐mesenchymal transition (EMT) process, evidenced by decreased E‐cadherin and increased N‐cadherin and Vimentin levels. These pro‐metastasis‐related phenotype effects induced by TFAP2A were effectively abrogated by the concurrent knockdown of SLC35F2. These data indicate that TFAP2A promotes LUAD migratory and invasive capacities primarily by upregulating SLC35F2.

Beyond migratory and invasive capacities, we further evaluated the impact of this regulatory axis on cell survival by focusing on the intermediate LncRNA NUTM2A‐AS1. We examined whether restoring SLC35F2 expression could rescue the apoptosis induced by NUTM2A‐AS1 silencing. As depicted in Figure 8I–K, knockdown of NUTM2A‐AS1 (sh‐NUTM2A‐AS1) promoted apoptosis, indicated by the marked upregulation of the pro‐apoptotic protein (Bax and cleaved Caspase‐3) and the downregulation of the anti‐apoptotic protein Bcl‐2. However, co‐transfection with the SLC35F2 overexpression plasmid (OE‐SLC35F2) successfully reversed these apoptotic molecular trends. Taken together, these rescue assays demonstrate that the integrated TFAP2A/NUTM2A‐AS1/SLC35F2 axis drives LUAD progression by simultaneously promoting metastasis‐related phenotypes and evading cell apoptosis.

## 4 Discussion

Lung adenocarcinoma (LUAD), accounting for approximately 40% of lung cancer cases, is frequently diagnosed at advanced stages, resulting in a poor prognosis and a 5‐year survival rate below 20%. Despite advances in surgical, chemotherapeutic and immunotherapeutic approaches, the rising incidence and high mortality rate of LUAD necessitate a deeper understanding of its molecular pathogenesis. Since LUAD exhibits distinct biological characteristics often managed under the broad umbrella of NSCLC, there is a scarcity of specific biomarkers. Therefore, elucidating the underlying mechanisms of LUAD is essential for discovering novel therapeutic targets. Our group has previously investigated NSCLC progression and therapeutic strategies from multiple perspectives, including immune checkpoints, metabolic remodelling and surgical management [33, 34, 35, 36]. Building upon these previous findings, the present study focuses on the function and regulatory network of the long non‐coding RNA NUTM2A‐AS1 in lung adenocarcinoma, aiming to reveal the role of the TFAP2A/NUTM2A‐AS1/miR‐409‐5p/SLC35F2 axis and provide new insights for molecular targeted therapy in lung adenocarcinoma.

The progression of LUAD is closely linked to aberrant gene expression, particularly in pathways governing cell proliferation and survival. Non‐coding RNAs (ncRNAs) have emerged as critical epigenetic regulators encoded within the genome. LncRNAs, in particular, are implicated in tumorigenesis and migratory and invasive capacities through their ability to interact with miRNAs and proteins. Acting as ceRNAs, LncRNAs can sequester miRNAs, thereby modulating the expression of downstream target genes involved in proliferation, apoptosis and migration [37, 38]. In this study, we constructed a LncRNA‐miRNA‐mRNA network and identified that LncRNA NUTM2A‐AS1 acts as a pivotal regulator in LUAD. We demonstrated that NUTM2A‐AS1 is upregulated in LUAD tissues under the transcriptional control of TFAP2A. Furthermore, we revealed that NUTM2A‐AS1 regulates SLC35F2 expression via a miR‐409‐5p‐dependent mechanism. Specifically, TFAP2A‐induced upregulation of NUTM2A‐AS1 promotes LUAD progression by acting as a ceRNA‐like regulator of miR‐409‐5p, which in turn modulates SLC35F2. These findings suggest that NUTM2A‐AS1 may serve as a valuable biomarker for prognosis and a potential therapeutic target. Our results align with emerging concepts in LncRNA biology regarding spatial regulation [39, 40]. For example, NUTM2A‐AS1 has been shown to influence cell viability via the miR‐590‐5p/METTL3 axis in LUAD [41] and via the miR‐126‐5p/FAM3C axis in colorectal cancer [42], highlighting the importance of its cytoplasmic localisation in oncogenic signalling.

TFAP2A is a member of the AP‐2 transcription factor family, which regulates proliferation and migration in various cancers [43, 44, 45]. In breast cancer, TFAP2A and TFAP2C are critical for maintaining specific subtypes; for instance, TFAP2C regulates luminal characteristics, while TFAP2A is essential for the basal subtype [46, 47, 48]. Despite exhibiting 83% structural resemblance to TFAP2C, TFAP2A has been reported to lack transcriptional function at promoters of luminal genes owing to its SUMOylation in luminal breast cancer cells. This modification hinders its capacity to stimulate luminal gene expression but serves a vital function in sustaining the basal BC subtype [43]. In ER‐positive BC, several investigations have indicated that TFAP2A enhances tumour advancement [49, 50]. Although TFAP2A has been implicated in LUAD development [29, 30], its precise mechanism remains incompletely understood. Our study provides novel evidence that TFAP2A is required for NUTM2A‐AS1‐mediated LUAD progression. We show that TFAP2A directly binds to the NUTM2A‐AS1 promoter, inducing its expression, which subsequently elevates SLC35F2 levels by sequestering miR‐409‐5p. By evaluating this top‐to‐bottom regulatory axis, we found that TFAP2A not only drives hyperproliferation but also regulates aggressive biological behaviours. Our rescue assays confirmed that TFAP2A‐induced migration, invasion and the epithelial‐mesenchymal transition (EMT) are at least partially mediated by SLC35F2. The ability of SLC35F2 knockdown to reverse the TFAP2A‐driven EMT signature highlights a functional coupling between oncogenic transcription and downstream metabolic/transporter targets in reshaping the tumour microenvironment.

SLC35F2, a nucleotide sugar transporter primarily located in the ER/Golgi, has been implicated in several malignancies [51, 52, 53, 54]. Elevated SLC35F2 expression correlates with pathological staging in NSCLC [55] and promotes aggressive phenotypes in papillary thyroid carcinoma [22] and bladder cancer [31]. In H1299 LC cells, SLC35F2 knockdown diminished proliferation, migration and invasion, further supporting its oncogenic role in LC [56]. Consistent with these reports, we found that SLC35F2 is upregulated in LUAD and acts as a downstream effector of the TFAP2A/NUTM2A‐AS1 axis. Our findings parallel those of He et al., who described a TFAP2A/TPRG1‐AS1 axis in bladder cancer [24] and Xiong et al., who reported a miR‐16/TFAP2A/PSG9 pathway in LUAD [30]. However, our study is the first to delineate the specific TFAP2A/NUTM2A‐AS1/miR‐409‐5p/SLC35F2 axis. The critical role of tumour‐suppressive miRNAs to restrain cell proliferation and metastasis is a universal phenomenon in oncology. Similar tumour‐suppressive roles have been reported for other miRNAs in distinct malignancies, such as miR‐124 in neuroblastoma [57]. Consequently, this persistent biochemical signalling regulates the downregulation of pro‐apoptotic proteins (Bax, Cleaved Caspase‐3) and drives the EMT to accelerate metastasis. Importantly, this molecular‐level metabolic reprogramming is highly consistent with macroscopic clinical observations. Previous studies have demonstrated that elevated metabolic parameters of primary tumours predict lymph node metastasis and poor clinical outcomes in patients with resectable NSCLC [58]. The reversal of these aggressive phenotypes upon SLC35F2 knockdown underscores its role as a metabolic‐signalling bridge in LUAD progression. While we have mapped this unidirectional pathway, potential feedback loops, such as SLC35F2 modulating TFAP2A via post‐translational modifications, warrant future investigation. Although our study firmly establishes the oncogenic role of the TFAP2A/NUTM2A‐AS1/SLC35F2 axis in LUAD, the precise biochemical mechanisms by which SLC35F2 drives proliferation warrant further investigation. As a member of the solute carrier family, SLC35F2 is primarily implicated in the transport of nucleotide sugars and other small molecules, which can influence metabolic remodelling and protein glycosylation. Altered glycosylation of receptor tyrosine kinases, driven by aberrant transporter activity, is a known mechanism for sustained proliferative signalling in cancer cells. However, a limitation of the current study is the absence of direct metabolomic profiling or substrate‐specific transport assays. Future studies employing mass spectrometry‐based metabolomics and intracellular ion flux analyses are necessary to map the exact metabolic pathways and intracellular microenvironment alterations regulated by SLC35F2 in LUAD. Compared to previous studies focusing on NUTM2A‐AS1 or TFAP2A individually, this study innovatively identifies a “transcription factor/ceRNA/solute carrier” cascade in LUAD. We demonstrate that the transcription factor TFAP2A activates LncRNA NUTM2A‐AS1, which subsequently sponges miR‐409‐5p to upregulate the oncogenic transporter SLC35F2. By integrating upstream transcriptional control with downstream SLC35F2‐driven malignant phenotypes (proliferation, metastasis and EMT), our research provides a novel mechanistic perspective on LUAD pathogenesis and identifies promising prognostic biomarkers and therapeutic targets. We acknowledge several limitations in our study that warrant further investigation. First, although we extensively validated the pro‐metastatic and EMT‐promoting roles of this axis using in vitro Transwell and biochemical assays, in vivo lung metastasis models (such as tail vein injection assays) are lacking and will be necessary to confirm these metastatic phenotypes in living organisms. Second, a fundamental limitation remains regarding the physiological relevance of the ceRNA hypothesis. The biological efficacy of ceRNA networks strictly depends on RNA stoichiometry. Because our interaction and rescue assays largely relied on exogenous transfections and viral transductions, the resulting artificially elevated RNA levels might overstate the actual endogenous sponging efficiency. Future studies utilising absolute stoichiometric tracking are required to rigorously validate the endogenous relevance of this axis. Third, the exact endogenous metabolite or nutrient transported by SLC35F2 in LUAD cells remains unidentified. Future trans‐omics studies integrating metabolomics and isotopic tracing are required to define the exact metabolic payload. Lastly, our clinical correlations and prognostic evaluations heavily relied on the TCGA database. Large‐scale, multi‐centre prospective cohorts with long‐term follow‐up are needed to independently validate the prognostic value of the TFAP2A/NUTM2A‐AS1/SLC35F2 axis before clinical application. Although not directly tested in the present study, recent work has suggested that combination strategies involving pathway modulators and bioactive compounds may enhance antitumor efficacy in lung cancer models [59]. This provides a rationale for future studies evaluating whether targeting the TFAP2A/NUTM2A‐AS1/SLC35F2 axis could be integrated into combinatorial therapeutic approaches.

## 5 Conclusion

In summary, this study highlights the oncogenic role of LncRNA NUTM2A‐AS1 in LUAD. We demonstrate that TFAP2A‐induced upregulation of NUTM2A‐AS1 promotes LUAD progression in vitro and in vivo by acting as a ceRNA‐like regulator of miR‐409‐5p and subsequently upregulating SLC35F2. These findings provide new insights into the molecular pathogenesis of LUAD and suggest that the TFAP2A/NUTM2A‐AS1/SLC35F2 axis represents a promising therapeutic target.

## Author Contributions

**Feng Hu:** conceptualization, investigation, writing – review and editing, resources, project administration, funding acquisition, data curation. **Jing Zhang:** conceptualization, investigation, writing – review and editing, validation, project administration, supervision, resources. **Weiqin Wang:** conceptualization, investigation, writing – original draft, methodology, validation. **Yongfeng Liu:** conceptualization, investigation, software, formal analysis, data curation, writing – original draft. **Tiantian Chen:** conceptualization, investigation, resources, visualization, writing – original draft.

## Funding

This work was supported by Shanghai Changning District Committee of Science and Technology of China Grant (CNKW2022Y05).

## Consent

Informed consent was obtained from all participants involved in the study. Patients provided written authorisation for the publication of this manuscript.

## Conflicts of Interest

The authors declare no conflicts of interest.

## Supporting information


**Figure S1:** Transfection efficiency validation results. (A) Relative mRNA levels of TFAP2A in A549, H358 and HCC827 cells after siRNA treatment. (B) Relative protein levels of TFAP2A in A549, H358 and HCC827 cells after siRNA treatment. (C) Representative western blot images of TFAP2A expression in A549, H358 and HCC827 cells after siRNA treatment. (D) Relative mRNA levels of TFAP2A in A549, H358 and HCC827 cells after overexpression treatment. (E) Relative protein levels of TFAP2A in A549, H358 and HCC827 cells after overexpression treatment. (F) Representative western blot images of TFAP2A expression in A549, H358 and HCC827 cells after overexpression treatment. (G, H) Relative levels of LncRNA NUTM2A‐AS1 in A549, H358 and HCC827 cells after siRNA or overexpression treatment. (I, J) Relative miR‐409‐5p levels after mimic or inhibitor treatment. (K, L) Relative mRNA levels of SLC35F2 in A549, H358 and HCC827 cells after overexpression or shRNA treatment. (M) Representative western blot images of SLC35F2 expression in A549, H358 and HCC827 cells after shRNA treatment. (N) Relative protein levels of SLC35F2 in A549, H358 and HCC827 cells after shRNA treatment. **p < 0.01 compared to Control, pcDNA3.1, mimic‐NC, inhibitor‐NC, OE‐NC or sh‐NC.

**Figure S2:** The levels of TFAP2A and SLC35F2 in normal lung epithelial cells and LUAD cell lines and the FACS profile of cells treated with sh‐NC alone. (A) The mRNA levels of TFAP2A and SLC35F2 in normal lung epithelial cells (BEAS‐2B) and in LUAD cell lines (A549, H358 and HCC827 cells) measured by qRT‐PCR. (B) The protein levels of TFAP2A and SLC35F2 in normal lung epithelial cells (BEAS‐2B) and LUAD cell lines (A549, H358 and HCC827 cells) measured by Western blot method. (C) Representative western blot images of TFAP2A and SLC35F2 expression in BEAS‐2B, A549, H358 and HCC827 cells. (D) Representative FACS images of Control HCC827 cells and HCC827 cells treated with sh‐NC alone and the cell apoptosis rate. **p < 0.01 compared to BEAS‐2B.

**Figure S3:** The levels and subcellular fractionation of NUTM2A‐AS1 and miR‐409‐5p in cells. (A–C) The subcellular fractionation of NUTM2A‐AS1 and miR‐409‐5p in LUAD cell lines (A549, H358 and HCC827 cells) (D–F) RIP assays confirmed the enrichment of both NUTM2A‐AS1 and miR‐409‐5p in AGO2 immunoprecipitates. (G, H) The relative quantification of NUTM2A‐AS1 and miR‐409‐5p in normal lung epithelial cells (BEAS‐2B) and in LUAD cell lines (A549, H358 and HCC827 cells). **p < 0.01 compared to BEAS‐2B.

**Table S1:** Antibodies used in the study.
**Table S2:** Primers used in the PCR procedure.

## Data Availability

The datasets used and/or analysed during the current study are available from the corresponding author on reasonable request.
