# Supplementary figures and images for: TFAP2A‐Induced Upregulation of LncRNA NUTM2A‐AS1 Promotes LUAD Progression Through a miR‐409‐5p/SLC35F2 Regulatory Axis
Source: J Cell Mol Med. 2026 Jul 10;30(13):e71284. doi: 10.1111/jcmm.71284 (PMC13354745; doi:10.1111/jcmm.71284)

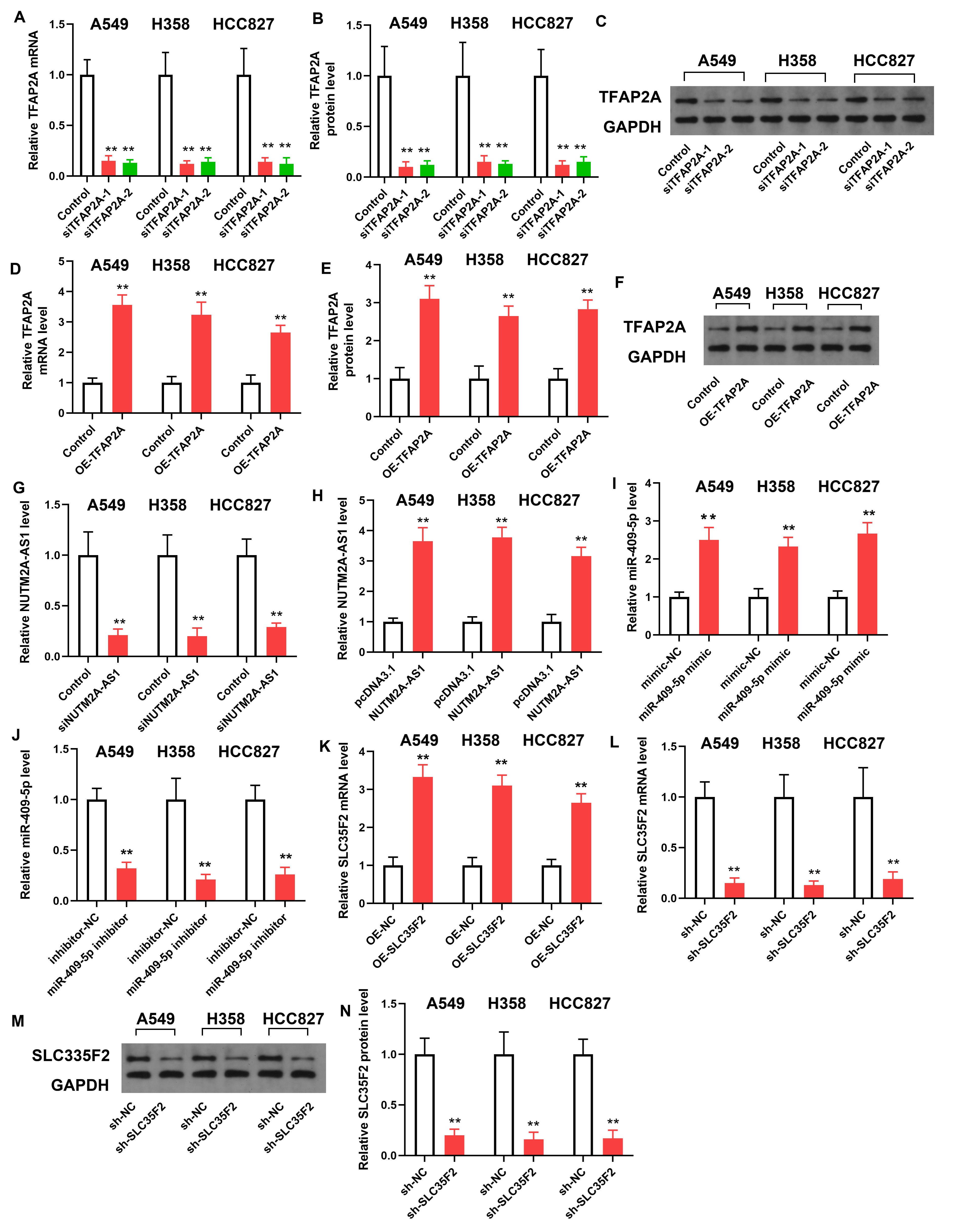

Supplement: Supplementary file 1 — Figure S1: Transfection efficiency validation results. (A) Relative mRNA levels of TFAP2A in A549, H358 and HCC827 cells after siRNA treatment. (B) Relative protein levels of TFAP2A in A549, H358 and HCC827 cells after siRNA treatment. (C) Representative western blot images of TFAP2A expression in A549, H358 and HCC827 cells after siRNA treatment. (D) Relative mRNA levels of TFAP2A in A549, H358 and HCC827 cells after overexpression treatment. (E) Relative protein levels of TFAP2A in A549, H358 and HCC827 cells after overexpression treatment. (F) Representative western blot images of TFAP2A expression in A549, H358 and HCC827 cells after overexpression treatment. (G, H) Relative levels of LncRNA NUTM2A‐AS1 in A549, H358 and HCC827 cells after siRNA or overexpression treatment. (I, J) Relative miR‐409‐5p levels after mimic or inhibitor treatment. (K, L) Relative mRNA levels of SLC35F2 in A549, H358 and HCC827 cells after overexpression or shRNA treatment. (M) Representative western blot images of SLC35F2 expression in A549, H358 and HCC827 cells after shRNA treatment. (N) Relative protein levels of SLC35F2 in A549, H358 and HCC827 cells after shRNA treatment. **p < 0.01 compared to Control, pcDNA3.1, mimic‐NC, inhibitor‐NC, OE‐NC or sh‐NC. [file JCMM-30-e71284-s004.tif]

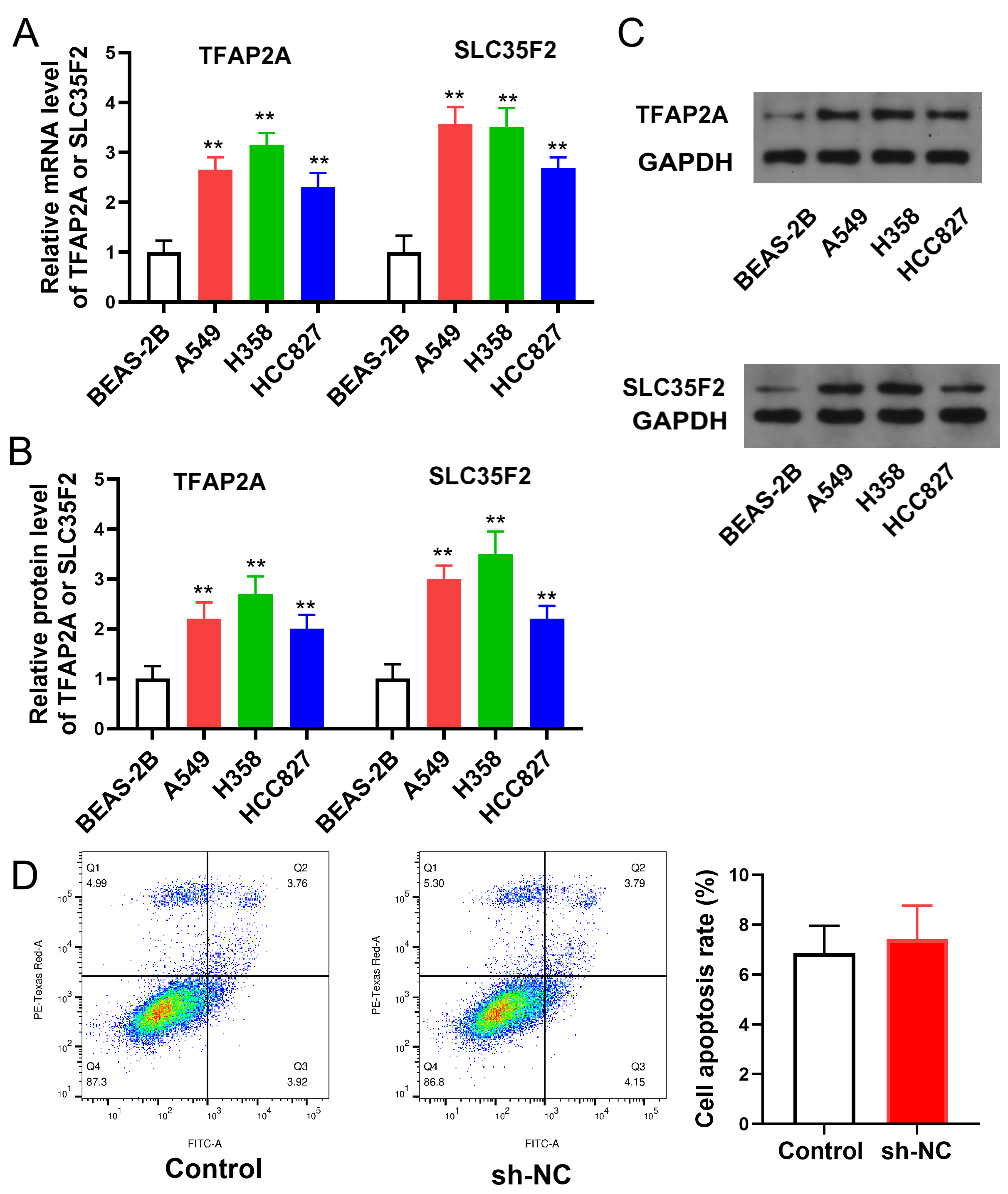

Supplement: Supplementary file 2 — Figure S2: The levels of TFAP2A and SLC35F2 in normal lung epithelial cells and LUAD cell lines and the FACS profile of cells treated with sh‐NC alone. (A) The mRNA levels of TFAP2A and SLC35F2 in normal lung epithelial cells (BEAS‐2B) and in LUAD cell lines (A549, H358 and HCC827 cells) measured by qRT‐PCR. (B) The protein levels of TFAP2A and SLC35F2 in normal lung epithelial cells (BEAS‐2B) and LUAD cell lines (A549, H358 and HCC827 cells) measured by Western blot method. (C) Representative western blot images of TFAP2A and SLC35F2 expression in BEAS‐2B, A549, H358 and HCC827 cells. (D) Representative FACS images of Control HCC827 cells and HCC827 cells treated with sh‐NC alone and the cell apoptosis rate. **p < 0.01 compared to BEAS‐2B. [file JCMM-30-e71284-s003.tif]

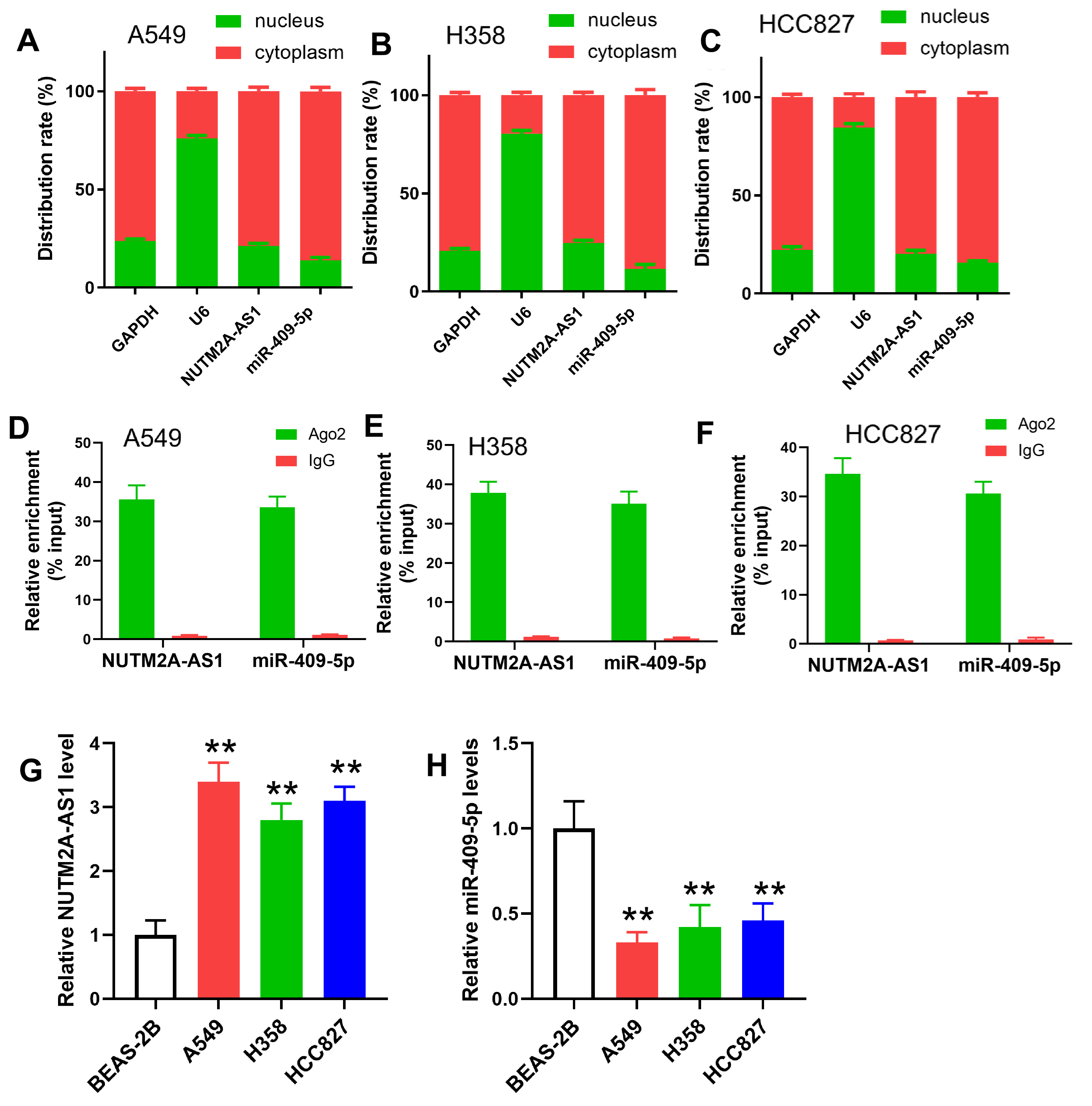

Supplement: Supplementary file 3 — Figure S3: The levels and subcellular fractionation of NUTM2A‐AS1 and miR‐409‐5p in cells. (A–C) The subcellular fractionation of NUTM2A‐AS1 and miR‐409‐5p in LUAD cell lines (A549, H358 and HCC827 cells) (D–F) RIP assays confirmed the enrichment of both NUTM2A‐AS1 and miR‐409‐5p in AGO2 immunoprecipitates. (G, H) The relative quantification of NUTM2A‐AS1 and miR‐409‐5p in normal lung epithelial cells (BEAS‐2B) and in LUAD cell lines (A549, H358 and HCC827 cells). **p < 0.01 compared to BEAS‐2B. [file JCMM-30-e71284-s002.tif]
