# Supplementary material for: TFAP2A‐Induced Upregulation of LncRNA NUTM2A‐AS1 Promotes LUAD Progression Through a miR‐409‐5p/SLC35F2 Regulatory Axis
Source: J Cell Mol Med. 2026 Jul 10;30(13):e71284. doi: 10.1111/jcmm.71284 (PMC13354745; doi:10.1111/jcmm.71284)
Supplement: Supplementary file 4 — Table S1: Antibodies used in the study. Table S2: Primers used in the PCR procedure. [file JCMM-30-e71284-s001.docx]

**Supplementary Table S1.** Antibodies used in the study.

| **Reagent** | **Source** | **Catalog No.** |
| --- | --- | --- |
| ***Primary antibodies*** | | |
| Anti-SLC35F2 antibody | Sigma-Aldrich | HPA040337 |
| Anti-TFAP2A antibody | Sigma-Aldrich | HPA055265 |
| Anti-CDK2 antibody | Abcam | ab10579 |
| Anti-CDK4 antibody | Abcam | ab155282 |
| Anti-CDK6 antibody | Abcam | ab125066 |
| Anti-cyclin E1 antibody | Novus | AF6810 |
| Anti-cyclin D1 antibody | Novus | NBP2-32840 |
| Anti-p21 antibody | Novus | NBP2-29463 |
| Anti-GAPDH antibody | Proteintech | 60004-1-Ig |
| Anti-Histone H3 antibody | Abcam | ab1791 |
| Anti-AGO2 antibody | Abcam | ab32381 |
| ***Secondary antibodies*** | | |
| Goat Anti-Rabbit IgG H&L (HRP) | Abcam | ab6721 |
| Goat Anti-Mouse IgG H&L (HRP) | Abcam | ab6789 |

**Supplementary Table S2.** Primers used in the PCR procedure.

| **Primers Source**  **Identifier** | | |
| --- | --- | --- |
| **Forward Reverse** | | |
| **CDK2** | GACACGCTGCTGGATGTCA | CAGAAAGCTAGGCCCTGGAG |
| **CDK4** | GTGTATGGGGCCGTAGGAAC | CCATAGGCACCGACACCAAT |
| **CDK6** | GAGCCGACTGACACTCGCA | CCTCCTCGAAGCGAAGTCC |
| **Cyclin E1** | AGAGGAAGGCAAACGTGACC | TTTGCCCAGCTCAGTACAGG |
| **Cyclin D1** | GAGGCGGAGGAGAACAAACA | GGAGGGCGGATTGGAAATGA |
| **P21** | AGTCAGTTCCTTGTGGAGCC | CATTAGCGCATCACAGTCGC |
| **SLC35F2** | CCTGCTGCGCAGGATAAAAG | TCTGAAGCATGGGGGTGTTC |
| **lncRNA NUTM2A-AS1** | TGCTGCTGTCAGTTTTGATGC | ACACGTTGCCTGTCTTTCTGA |
| **GAPDH** | ATTTCTCCTCCGGGTGATGC | CTTCCCGTTCTCAGCCTTGA |
